# Biomechanical effect of unloader braces for medial osteoarthritis of the knee: a systematic review (CRD 42015026136)

**DOI:** 10.1007/s00402-015-2388-2

**Published:** 2016-01-06

**Authors:** Wolf Petersen, Andree Ellermann, Thore Zantop, Ingo Volker Rembitzki, Hartmut Semsch, Christian Liebau, Raymond Best

**Affiliations:** Klinik für Orthopädie und Unfallchirurgie, Martin Luther Krankenhaus, Caspar Theysstr 27-31, 14193 Berlin, Germany; Arcus Sportklinik, Pforzheim, Germany; Sporthopaedicum, Straubing, Germany; Deutsche Sporthochschule, Cologne, Germany; Ortema, Markgröningen, Germany; Asklepios Klinikum Bad Harzburg, Bad Harzburg, Germany; Sportklinik Stuttgart, Stuttgart, Germany

**Keywords:** Knee adduction moment, Medial osteoarthritis, Varus malalignement, Biomechanics

## Abstract

**Purpose:**

There is a lack of consensus regarding biomechanical effects of unloader braces for the treatment of medial osteoarthritis (OA) of the knee. The purpose of this study was to perform a systematic review of studies examining the biomechanical effect of unloader braces.

**Methods:**

A systematic search for articles about the biomechanical effect of unloader braces was performed. Primary outcome measure was the influence of the brace on the knee adduction moment. Data sources were Pubmed central and google scholar.

**Results:**

Twenty-four articles were included. Twenty articles showed that valgus unloader braces significantly decrease the knee adduction moment. Seven of those studies reported a decrease of pain in braced patients (secondary outcome measure). Positive effects on the knee adduction moment could be found for custom made braces for conventional knee braces and for a foot ankle orthosis. Four studies could not show any effect of knee unloader braces on the knee adduction moment although one of these studies found decreased pain in braced patients. One of these studies examined healthy patients with a neutral axis.

**Conclusion:**

This systematic review could demonstrate evidence that unloader braces reduce the adduction moment of the knee. Foresighted, a systematic review about the clinical effect of unloader braces is required.

## Introduction

Osteoarthritis (OA) is a frequent cause of knee pain especially in the elderly population. The prevalence of OA is expected to increase as the population ages [33]. The initial treatment of OA is non-operative and consists of patient education, weight reduction, physical therapy, and pain relieving medication [27].

In knee joints with varus malaligement, the ground reaction force vector runs medially and from the middle of the knee baseline. The moment arm is the perpendicular distance between the ground reaction force vector and the knee’s center of rotation. This moment arm produces an external adduction moment, also named varus moment [33]. The knee adduction moment has been identified as the mechanism primarily responsible for the increased compressive load on the medial compartment of varus knees [33]. A large adduction moment shifts the weight bearing line medially within the knee and leads to increased medial compartment loading [33].

Consequently, especially in unicompartimental OA, knee alignment plays an important role for disease progression and functional decline [38]. Besides the possibilities of surgical axis correction procedures, non-surgical interventions for unicompatimental OA are knee braces which may alter the alignment of the lower extremity [2, 27, 33]. These so-called unloader braces apply an external varus or valgus force to the knee to shift loads towards the non-affected compartment [33]. E.g. the external valgus force acts via condylar pads or straps while opposing counterforces which arise from the supports proximal and distal to the knee joint [33]. Due to the high prevalence of medial OA, valgus bracing is used more frequently than varus bracing.

Randomized controlled trials (RCT) could confirm that in patients with medial OA and varus malalignment knee bracing—in comparison to non-treated patients—results in improved knee function [3, 40]. However, there is still a debate about the exact biomechanical effect of unloader braces [4].

For example, Horlick and Loomer [17] found no significant influence of an unloader brace on the femoral-tibial angle or joint space. In contrast, Komistek et al. [21] demonstrated significant condylar separation of the medial compartment with the use of valgus bracing. These conflicting results may result from differences in study or brace design.

The goal of this article was to perform a systematic literature review of biomechanical studies investigating the effect of unloader braces. Because of its biomechanical significance, particular attention was given to the reduction of the adduction moment. Regarding the outcome, we hypothesized that unloader braces are able to reduce the adduction moment of the knee.

## Methods

### Search details

We conducted a comprehensive literature search using the Pubmed database and Google scholar to identify peer reviewed articles about the biomechanical effects of knee unloader braces used for the treatment of medial OA according to the PRISMA statement [15, 28]. The PRISMA Statement consists of a 27-item checklist and a four-phase flow diagram [15, 28].

Before the literature search, the study was registered at PROSPERO which is an international database of prospectively registered systematic reviews (http://www.crd.york.ac.uk/PROSPERO). The registry number of this systematic review is CRD42015026136.

For the systematic review different combinations of keywords were utilized: (1) Valgus brace, (2) Knee osteoarthritis and brace, (3) Knee adduction moment. When a study of interest was found, related articles were searched. Time frame for the search was September 15th to October 30th 2015. After identifying the articles, all references were screened for additional relevant publications.

### Inclusion and exclusion criteria

Inclusion criteria for this systematic review were:

1. Biomechanical study about unloader braces
2. Measurement of the knee adduction moment as outcome criterion
3. English language reports, and
4. Publication in a peer reviewed journal.

Exclusion criteria were:

1. Number of study participants <5
2. Cadaveric study
3. Systematic reviews or meta-analyses

The abstract of each relevant article was checked. In case of a mismatch with one of the inclusion criteria or match with the exclusion criterion the study was excluded. In case of an eligible article, the full text of the original article and the published appendices as well as the previously, elsewhere published study protocols were studied.

Two reviewers (WP, RB) performed the initial study identification, secondary study screening, and final determination of eligibility and study inclusion. Each of the two reviewers was also involved in the analysis of the articles.

The primary research question reviewing the articles was: Is there any effect of valgus unloader braces on the knee adduction moment?

### Analysis

After extraction of all study data, a brief tabular narrative of each investigation was presented. Data of these tables include (1) references, (2) number of patients, brace type, methods, disease, (3) primary outcome (Tables 1, 2). Additional tables were added to illustrate other results of the included studies (Tables 3, 4).

**Table 1:** Biomechanical studies about valgus unloader braces

References	Number, brace, methods, disease	Primary outcome measure
Arazpour et al. [1]	7 patients, conventional valgus brace, gait analysis, patients with medial OA	The knee adduction moment was significantly reduced (*p* = 0.001)
Della Croce et al. [4]	18, pneumatic unloading knee brace, gait analysis, patients with medial OA	A 7.6 % decrease in net peak knee adduction moment with the brace uninflated and 26.0 % with the brace inflated
Dessery et al. [5]	24, custom valgus knee brace (three point bending brace, valgus and external rotation brace, ACL brace), gait analysis, patients with medial OA	The valgus unloader brace and ACL-brace allowed a significant reduction in peak knee adduction moment (KAM) during terminal stance from 0.313 to 0.280 Nm/Bw × Ht (*p* < 0.001) and 0.293 to 0.268 (*p* < 0.05) respectively, while no significant reduction was observed with the V3P-brace (*p* = 0.52)
Draganich et al. [6]	10, conventional brace, custom brace, gait analysis, patients with medial OA	The custom-brace significantly decreased peak adduction moments during gait and stair stepping, compared with baseline and off-the-shelf bracing
Fantini Pagani et al. [9]	16, conventional valgus unloader brace, gait analysis, healthy volunteers	The first and second peak knee adduction moments also decreased during walking with different orthosis adjustments (changes from 5 to 33 %). During running, a significant reduction was observed only between the conditions without orthosis and 8° valgus adjustments (change of 11 %)
Fantini Pagani et al. [10]	10, conventional valgus brace, gait analysis, patients with medial OA	For the second peak knee adduction moment, decreases of 18, 21, and 7 % were observed between baseline and test conditions for the orthosis in 4° valgus, in 8° valgus, and insole, respectively.
Fantini Pagani et al. [11]	14, ankle–foot orthosis, gait analysis, healthy subjects with knee varus alignment	Significant decreases in knee adduction moment, in the frontal plane were observed with the ankle–foot orthosis in all three different adjustments
Fu et al. [13]	10, conventional valgus knee brace, gait analysis, patients with medial OA	Gait analysis indicated statistically significant reductions in peak and mean knee adduction moments in all orthotic groups when compared with a flat insole
Johnson et al. [19]	10, conventional valgus brace, gait analysis, patients with medial OA	The mean improvement in knee adduction moment was a decrease of 0.2255 Nm/kg (range 0.56–0.564 Nm/kg), showing a mean improvement of 48 % (range 16–76 % of original peak moment)
Jones et al. [19]	28, conventional valgus brace, gait analysis, patients with medial OA	The valgus knee brace, reduced the early stance EKAMexternal knee adduction moment by 7 %
Laroche et al. [24]	20, valgus and external rotation brace, gait analysis, patients with medial OA	Knee adduction moments significantly decreased in the terminal stance and push off
Lamberg et al. [23]	15, conventional valgus knee brace, gait analysis, patients with medial OA	Second peak knee adduction moment were reduced (*p * < 0.05) at post and final compared to baseline (26 %)
Lindenfeld et al. [26]	11, custom valgus knee brace, gait analysis, patients with medial OA	9 of 11 patients had a decrease in the adduction moment when wearing the brace, with the moment decreasing by as much as 32 %
Moyer et al. [29]	16, custom valgus knee brace, gait analysis, patients with medial OA	Valgus bracing reduced knee adduction moment. The reduction in knee adduction moment was greatest when using the knee brace and a foot orthotic
Orishimo et al. [30]	12, conventional valgus knee brace, gait analysis, normally aligned patients	Peak knee adduction moment and knee adduction impulse decreased with increasing brace tension (main effect of brace, *p* < 0.001)
Ota et al. [31]	15, custom made valgus brace, gait analysis, patients with medial OA	The peak KAM with KBF was significantly smaller than those with the KB (*p* = 0.004, the difference between these conditions of KAM: 0.06 Nm/kg)
Pollo et al. [32]	11, conventional valgus knee brace, gait analysis, patients with medial OA	Valgus bracing reduced the adduction moment about the knee by an average of 13 % (7.1 Nm)
Ramsey et al. [34]	16, custom valgus knee brace, gait analysis, patients with medial OA	Knee adduction excursions were significantly reduced with the use of bracing, with excursions reported to be lowest at 4° of valgus correction
Self et al. [35]	5, custom made valgus knee brace, gait analysis, patients with medial OA	The Monarch brace significantly reduced the varus moment at 20 and 25 % of stance
Schmalz et al. [36]	16, conventional valgus brace, gait analysis, patients with medial OA	The mean maximum value of the orthotic valgus moment was 0.053 Nm/kg, which represents approximately 10 % of the external genu varus moment without the brace

**Table 2:** Biomechanical studies showing no effects of valgus unloader braces on the knee adduction moment

References	Number, brace, methods	Primary outcome measure
Duivenvoorden et al. [7]	80, conventional knee unloader brace, gait analysis, patients with medial OA	No reduction of knee adduction moment, in the brace group at baseline and after 6 weeks
Ebert et al. [8]	20, conventional knee unloader brace, gait analysis, normally aligned knees	Valgus bracing increased knee adduction moments
Gaasbeek et al. [14]	15, conventional knee unloader brace, gait analysis, patients with medial OA	Gait analysis showed that the brace had a tendency of lowering peak varus moment about the knee. This effect was more profound in the presence of higher initial varus deformity angle of the knee
Hewett et al. [16]	18, conventional knee unloader brace, gait analysis, patients with medial OA	Results while wearing a brace showed no significant change in the adduction moment

**Table 3:** Secondary outcomes of biomechanical studies which showed that valgus unloader braces decrease the knee adduction moment

References	Secondary outcome measures
Arazpour et al. [1]	Speed of walking significantly increased (*p* < 0.001) Reduction in knee range of motion (*p* = 0.002) Increase in step length (*p* < 0.001)
Dessery et al. [5]	Knee pain was alleviated with all three braces (*p* < 0.01)
Fantini Pagani et al. [11]	Significant decreases in knee lever arm, and joint alignment in the frontal plane were observed with the ankle–foot orthosis in all three different adjustments. No significant differences could be found in any parameter while using the laterally wedged insoles
Fu et al. [13]	Compared with pretreatment, the lateral-wedged insole, lateral-wedged insole with arch support, and valgus knee brace groups demonstrated significant reductions in WOMAC pain score (19.1 %, *p* = 0.04; 18.2 %, *p* = 0.04; and 20.4 %, *p* = 0.02, respectively). The valgus knee brace with lateral-wedged insole with arch support group demonstrated an additive effect with a statistically significant reduction in WOMAC total score (−26.7 %, *p* = 0.01). Compliance with treatment for the isolated insole groups were all over 90 %, but compliance for the valgus knee brace-associated groups was only around 50 %
Johnson et al. [18]	All but one of the compliant patients reported a decrease of at least two pain points after 3 months of use. There was one additional intervention in the brace cohort versus a statistical increase of 10 in the nonbrace cohort. All patients who were compliant with the brace showed an increase in thigh girth measurements, compared with none in the nonbrace cohort. Braced patients experienced retained improvements in at least one gait parameter including improved walking speed, total range of motion, and improved knee-angle at heel strike
Jones et al. [19]	Lateral wedged insole significantly increased walking speed, reduced the early stance EKAM 12 %, and the knee adduction angular impulse by 8.6 and 16.1 % respectively. The lateral wedged insole significantly reduced the early stance EKAM compared to the valgus knee brace (*p* = 0.001). The valgus knee brace significantly reduced the knee varus angle compared to the baseline and lateral wedged insole. Improvements in pain and function subscales were comparable for the valgus knee brace and lateral wedged insole. There were no significant differences between the two treatments in any of the clinical outcomes; however the lateral wedged insoles demonstrated greater levels of acceptance by patients
Laroche et al. [24]	VAS-pain and WOMAC significantly decreased at W5. Walking speed was not significantly modified by knee bracing at W0, but increased significantly at W5
Lindenfeld et al. [26]	No apparent gait adaptations were observed. Scores from an analog pain scale decreased 48 % with brace wear, and function with activities of daily living increased 79 %
Orishimo et al. [30]	With increasing tension in the brace, peak frontal plane knee angle shifted significantly from 1.6° ± 4.2° varus without the brace to 4.1° ± 3.6° valgus with maximum brace tension
Ota et al. [31]	The peak knee flexion angles during swing phase with KBF were also significantly larger than those with the KB (*p* = 0.004, the difference between these conditions of knee flexion angle: 1.5°)
Pollo et al. [32]	The medial compartment load at the knee decreased by an average of 11 % (114 N) Pain and activity level improved in all subjects with valgus bracing
Ramsey et al. [34]	Knee function and stability scored best with the brace in the neutral setting compared with the brace in the valgus setting. The cocontraction of the vastus lateralis-lateral hamstrings was significantly reduced from baseline in both the neutral (*p* = 0.014) and valgus conditions (*p* = 0.023), and the cocontraction of the vastus medialis-medial hamstrings was significantly reduced with the valgus setting (*p* = 0.068), as a result of bracing. Patients with greater varus alignment had greater decreases in vastus lateralis-lateral hamstring muscle cocontraction
Schmalz et al. [36]	Use of the tested brace also decreased the magnitude of gait asymmetry between the braced and contralateral legs during walking (horizontal ground reaction force, external knee flexion moment), presumably because the subjects’ need to walk abnormally to shield the knee from pain was reduced

**Table 4:** Secondary outcome measures of biomechanical studies showing no effects of valgus unloader braces on the knee adduction moment

References	Secondary outcome measures
Duivenvoorden et al. [7]	No reduction ground reaction force was seen in the brace group at baseline and after 6 weeks
Gaasbeek et al. [14]	Bracing led to a small decrease in knee extension at the end of the swing phase and increase of walking velocity
Hewett et al. [16]	Before brace wear, 78 % had pain with activities of daily living, but after the first evaluation, only 39 % continued to have such pain, and at the second evaluation, only 31 % were so affected. Before brace wear, patients had a walking tolerance of 51 min prior to the onset of pain symptoms. At the first evaluation, patients could walk 138 min without pain, and after 1 year, they could walk 107 min without pain. Before brace wear, 78 % rated their overall knee condition as fair or poor whereas at the first evaluation, only 33 % continued to provide this rating

### Primary, secondary and tertiary outcome measures

The following primary endpoint was analyzed: measurement of the knee adduction moment. Secondary endpoints are parameters with clinical relevance such as pain, function (scores) or gait parameters such as gait symmetry, step length and walking speed. Tertiary endpoints are all other findings reported in the extracted studies.

## Results

### Search results

**Fig. 1:**
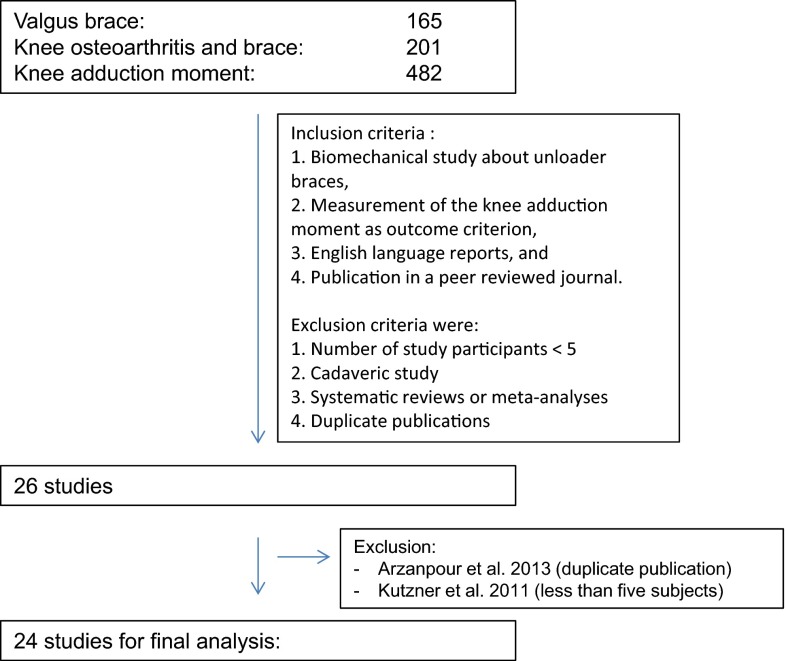
Flowchart showing the literature review

The article search results are shown in Fig. 1. Of all identified relevant articles, 24 original articles matched the inclusion criteria [1, 4–11, 13, 14, 16, 18, 19, 22–24, 26, 29–32, 34–36].

### Primary outcome measure

The primary outcome measure was the influence of the brace on the knee adduction moment (Tables 1, 2).

Twenty articles could be found which showed that valgus bracing decreased the knee adduction moments (Table 1). Three different brace designs were examined: custom made knee unloader braces, conventional knee unloader brace, and a foot ankle orthosis. Positive effects on the adduction moment could be found for all three brace types. Sixteen studies found a positive effect of patients with varus malalignement and medial OA [1, 4–6, 10, 13, 18, 19, 23, 24, 26, 29, 31, 32, 35, 36]. One study found an effect of unloader braces on knee adduction moment in patients with a normally aligned knee [30]. Two studies examined healthy subjects with knee varus alignment [9, 11]. In one study a neutral brace was as effective as a valgus brace [34].

Four studies could not show any effect of knee unloader braces on the knee adduction (Table 2). One of these studies examined healthy patients with a neutral axis [8]. In one of these studies there was a tendency of lowering peak varus moment, but the difference was not significant [14].

### Secondary outcome measures

Tables 3 and 4 summarise the included articles contents respecting secondary outcome measures. The most frequent reported secondary outcome measure was pain. Nine studies reported a decrease of pain in braced patients [5, 13, 16, 18, 19, 24, 26, 32, 36]. One of those studies showed no significant change in the adduction moment [16].

Three studies could show that valgus bracing increased gait speed [1, 14, 26] and two studies could show that valgus bracing increased step length [1, 26]. One study found that use of a valgus brace reduced gait asymmetry between the braced and contralateral legs during walking [36]. One study could not demonstrate any effects of bracing on gait parameters [26].

### Tertiary outcome measures

Three studies compared valgus bracing with laterally wedged insoles [10, 13, 19]. Fantini Pagani et al. [10] found no effect in knee lever arm, and joint alignment in the frontal plane while using the laterally wedged insoles. Jones et al. [19] could show that lateral wedged insoles significantly increased walking speed, reduced the early stance knee adduction moment, and the knee adduction angular impulse by 8.6 and 16.1 % respectively. In this study the lateral wedged insole could significantly reduce the early stance knee adduction moment compared to the valgus knee brace. Improvements in pain and function subscales were comparable for the valgus knee brace and lateral wedged insole. There were no significant differences between the two treatments in any of the clinical outcomes; however the lateral wedged insoles demonstrated greater levels of acceptance by patients compared to brace treatment. Similar results are reported by Fu et al. [12]. In this study, the lateral-wedged insole, and valgus knee brace groups demonstrated significant reductions in WOMAC pain score [12]. Compliance with treatment for the isolated insole groups were all over 90 %, but compliance for the valgus knee brace-associated groups was only around 50 % [12].

Other parameters which were positively affected by bracing were the foot progression angles (decreased in the terminal stance and push off) or lower-limb joint angles, moments and power [24]. One study found that co-contractions of the vastus lateralis-lateral hamstrings was significantly reduced by neutral and valgus bracing, and the co-contractions of the vastus medialis-medial hamstrings was significantly reduced by valgus bracing. Patients with greater varus alignment had greater decreases in vastus lateralis-lateral hamstring muscle co-contraction [34].

## Discussion

Our systematic review could clearly show that the majority of biomechanical studies confirmed a reduction of knee adduction moment by valgus bracing. These findings support our initial hypothesis.

However, the amount of reduction varied between the several studies. One study revealed a decrease of knee adduction moment in up to 32 % [26]. Of course, this finding could have clinical relevance as Kemp et al. [20] have shown that already 20 % more peak adduction moment can increase the risk of OA progression.

On the other hand, Pollo et al. [32] could demonstrate a knee adduction moment reduction of only 11 %. Even with this lower decrease in all subjects pain and activity level improved with valgus bracing [32].

Only four studies could not find any effect of unloader braces on the knee adduction moment [7, 8, 14, 16]. Hewett et al. [16] and Duivenvorden [7] found no effect of valgus bracing on knee adduction moment in patients with medial OA. Gaasbeek et al. [14] found valgus bracing tended to lower peak adduction moments, although differences were not statistically significant. Ebert [8] found increased knee adduction moments of unloader braces in patients with normally aligned knees.

Causes for these contradictory results can be differences in brace design, patient characteristics, the power of the different studies, or the methods used in the studies. For example Gaasbeck et al. [14] could show that in patients with greater varus knee deformity, the effect of bracing was more profound, with greater reductions in knee adduction moment. Lindenfeld et al. [26] showed that in 2 of 11 patients valgus bracing had no effect on knee adduction moment although the overall decrease in this study was 10 %. Unfortunately the patient characteristics of those patients were not described in the included studies. Komistek et al. [21] reported that among obese patients, a lack of subjective pain relief was noted and correlated with the absence of condylar separation in braced patients. In conclusion, further studies are needed to characterize the OA patients who are the ideal candidates for brace treatment.

Also in terms of pain, the results of the included studies were more than pointing the way. Although Hewett et al. [16] could not find any significant effect of valgus bracing on the knee adduction moment pain relief and extended walking time after wearing the brace was reported in this study. This finding clearly indicates that also other brace effects than reducing the knee adduction moment may be relevant. One of these effects could be the stabilizing function of the braces.

It is well known that knee OA is associated with frontal plane and medial–lateral joint laxity or instability [12, 25, 37]. In OA patients, joint laxity and instability is compensated by increased muscular co-contraction [34]. In addition, Ramsey et al. [34] could show that valgus and neutral bracing reduced pain and disability. Either bracing condition significantly reduced knee adduction moment but also muscle co-contractions. These results indicate that diminished muscle co-contractions may contribute to the effect of knee braces on pain relief in patients with medial OA.

**Fig. 2:**
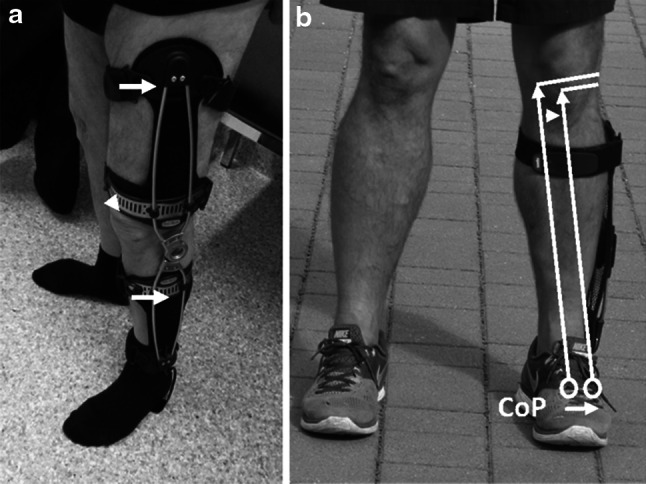
**a** Knee unloader brace with a unilateral hinge. The external valgus force acts via the condylar pas while opposing counterforces which arise from the supports proximal and distal to the knee joint (*arrows*). **b** Foot ankle orthosis (Aegillium free step, Otto Bock, Duderstadt). The lateral support reduces the valgus angle lateralizing the center of pressure (CoP)

Positive effects regarding their influence on the knee adduction moment could be detected for all brace designs: custom made, off the self, braces with condylar pads, braces with sleeves and foot ankle orthosis (Fig. 2). However, there is some evidence in the literature that some braces are more effective than others. For example, Draganich et al. [6] observed that reductions in the knee adduction moments were 3 to 4 times greater for custom braces than for off-the shelf braces. Causative for this observation might be the better fit of custom made braces. Another factor may be the amount of valgus angulation. Pollo et al. [9] and Fantini Pagani et al. [32] could show that adjusting valgus angulation from 4° to 8° had a significant effect on reducing the medial compartment load.

Not least, activity seemed to have also an effect on load reduction by valgus bracing. During walking, Fantini Pagani et al. [9] could reveal reductions of 25 and 36 % in the knee adduction angular impulse (4 and 8° valgus). Mean reductions of 18 and 23 % were still observed whilst running [9].

A new unloader brace design was examined by Fantini Pagani et al. [11]. Here an ankle–foot orthosis was used to reduce the knee adduction moment. This knee OA ankle brace (Aegillium free step, Otto Bock, Duderstadt, Germany) consists of a non-flexible insole which is connected to a lever arm with a pad applying a valgus force to the thigh. A possible advantage of this brace concept could be a lower rate of skin irritations due to the lack of condylar pads as to date several studies [3, 39, 40] have shown that skin irritations are a frequent cause for discontinuing brace therapy. Further prospective randomized studies are needed to assess the clinical effect of the named foot ankle orthosis.

A limitation of the present review is that that the quality of the included studies was not assessed or graduated respectively. However, to our knowledge, a quality assessment tool for biomechanical studies does not exist.

In conclusion, this systematic review could demonstrate evidence that valgus bracing can unload the medial compartment in patients with medial OA by reduction of the knee adduction moment. Beyond this this systematic review could reveal other effects of valgus bracing than reduction of the knee adduction moment. The most important clinical effect was pain reduction [5, 13, 24, 32, 36]. Other biomechanical effects include increased walking speed, increase in step length or increased gait symmetry [1, 18, 36].
